# YOLOF-Snake: An Efficient Segmentation Model for Green Object Fruit

**DOI:** 10.3389/fpls.2022.765523

**Published:** 2022-06-09

**Authors:** Weikuan Jia, Mengyuan Liu, Rong Luo, Chongjing Wang, Ningning Pan, Xinbo Yang, Xinting Ge

**Affiliations:** ^1^School of Information Science and Engineering, Shandong Normal University, Jinan, China; ^2^Key Laboratory of Facility Agriculture Measurement and Control Technology and Equipment of Machinery Industry, Zhenjiang, China; ^3^School of Light Industry Science and Engineering, Qilu University of Technology, Shandong Academy of Sciences, Jinan, China; ^4^China Academy of Information and Communications Technology, Beijing, China; ^5^School of Medical Imaging, Xuzhou Medical University, Xuzhou, China

**Keywords:** automatic harvesting, green fruits, YOLOF-snake, deep-snake, fruits segmentation

## Abstract

Accurate detection and segmentation of the object fruit is the key part of orchard production measurement and automated picking. Affected by light, weather, and operating angle, it brings new challenges to the efficient and accurate detection and segmentation of the green object fruit under complex orchard backgrounds. For the green fruit segmentation, an efficient YOLOF-snake segmentation model is proposed. First, the ResNet101 structure is adopted as the backbone network to achieve feature extraction of the green object fruit. Then, the C5 feature maps are expanded with receptive fields and the decoder is used for classification and regression. Besides, the center point in the regression box is employed to get a diamond-shaped structure and fed into an additional Deep-snake network, which is adjusted to the contours of the target fruit to achieve fast and accurate segmentation of green fruit. The experimental results show that YOLOF-snake is sensitive to the green fruit, and the segmentation accuracy and efficiency are significantly improved. The proposed model can effectively extend the application of agricultural equipment and provide theoretical references for other fruits and vegetable segmentation.

## Introduction

With the increasing maturity of artificial intelligence technology, new enlightenment is brought in fruit and vegetable production management and automated picking. The level of production automation (Bac et al., 2014; Jia et al., 2020c) and management intelligence (Bochtis et al., 2014; Caicedo Solano et al., 2020) is increased in the fruit and vegetable industry. The operating performance of vision systems directly affects the operational efficiency of agricultural equipment. It plays a vital role in the efficient production of fruits and vegetables (Patrício and Rieder, 2018; Tang et al., 2020; Tian et al., 2020), so achieving accurate segmentation and recognition of the fruit of an object becomes the key to precise yield measurement and automatic harvesting operations in orchards (Koirala et al., 2019; Van Klompenburg et al., 2020). However, due to the interference of branches and leaves, overlapping fruits, light and weather changes, operating angles, and the impact of the same color background of green fruit in the complex orchard environment, the efficient and accurate segmentation of green fruit is subject to many challenges, which attracts the attention of many scholars.

In previous research on object fruit segmentation and recognition, traditional machine learning has played a pivotal role in driving the development of intelligent agriculture (Jha et al., 2019; Sharma et al., 2020; Benos et al., 2021; Tahaseen and Moparthi, 2021). For example, Zhang et al. (2020) proposed a segmentation method combining color and texture features, using grayscale co-occurrence matrix (GLCM) to extract texture features. The recognition accuracy of the new method reached 0.94, but the process is affected by illumination and is not effective in segmenting the fruit of the object under uneven illumination. Besides, the darker area is not clearly segmented. Henila et al. (2020) constructed a fuzzy clustering-based threshold segmentation method for segmenting regions of interest in apple images with the largest cluster of pixels to calculate the threshold value, which has substantially improved the segmentation accuracy and efficiency compared with the grayscale thresholding method. Jia et al. (2020a) proposed an apple image recognition method based on PCNN and GA–Elman fusion. First, PCNN is utilized to implement segmentation of the target image, extract color, and shape features, GA–Elman classifier is designed, and finally, object fruit recognition is implemented. Aiming at the problem of apple fruit recognition with uneven coloring, Liu et al. (2019) proposed a target fruit segmentation algorithm based on superpixel features. The target image is segmented into pixel blocks by SLIC, and the target fruit and background are divided into two categories by SVM. And then, the classification results are further revised according to the adjacency relationship between superpixels. For the shadow problem of strongly illuminated images, Xu et al. (2019) proposed the method of fusing superpixels and edge probability maps to generate superpixel blocks with precise boundaries and using the relighting method to eliminate undetected shadows, which has strong robustness. However, the model training is time-consuming and the process is tedious. These methods have achieved relatively good recognition accuracy and efficiency and provide necessary theoretical and technical support for orchard yield measurement and machine picking (Pallathadka et al., 2021). However, most of these methods are studied for some specific scenarios and overly rely on the fruit color, shape, texture, and other features of the target fruit, which are greatly affected by light, resulting in poor robustness of the algorithms. In complex orchard environments, the extraction of target fruit features poses further difficulties for this identification method and does not meet the needs of agricultural equipment working under challenging situations.

In recent years, with the rapid development of deep learning and computing technology, deep learning algorithms have the advantages of end-to-end automatic detection and deep extraction of image features. Their robustness and accuracy have greatly improved, which are widely used in the fields of object detection and image segmentation (Zhao et al., 2019; Oksuz et al., 2020; Minaee et al., 2021). Inspired by this, deep learning has been increasingly used in agricultural production, such as pest and disease identification (Ushadevi, 2020; Liu and Wang, 2021), fruit and vegetable yield measurement (Yin et al., 2020; Maheswari et al., 2021), and automatic harvesting (Saleem et al., 2021; Yin et al., 2021), which substantially promote the development of agricultural technology. For complex environments, the research of green object fruit recognition based on deep learning theory has attracted the attention of many scholars, such as Farkhani et al. (2021), used a transformer-based multilayer attention procedure combined with fusion rules to accurately classify weeds by high-resolution attention maps, obtaining high accuracy results. Dhaka et al. (2021) studied and reviewed the existing models on the application of deep convolutional neural networks to predict plant diseases and insect pests from leaf images and compared the advantages and disadvantages of different technologies and models. Kundu et al. (2021) proposed an automatic and intelligent data collector and classifier framework that inherited the Internet of Things and deep learning technology. It will send the collected data to the cloud server and use Raspberry Pi to accurately predict the blast and rust diseases in pearl millet. Its classification accuracy can be comparable to the most advanced model, but it reduces the time by 86.67% (Kundu et al., 2021). Woźniak and Połap (2018) proposed developed neural network architecture, AANN, which processes relatively more miner information and makes numerical calculations more accurate. The proposed fusion is effective in the system structure and the training process on classification results (Woźniak and Połap, 2018). Li et al. (2021) optimized the U-Net model by combining the spatial pyramid pooling (ASPP) structure and merging U-Net’s edge features and advanced functions. In addition, this model obtained the semantic boundary information of object fruit images by integrating the residual module and closed convolution, which effectively improved the segmentation accuracy of the object fruit (Li et al., 2021). Xiong et al. (2020) used the YOLOv2 model to detect green mango images based on mango images collected by UAVs, and the detection accuracy is only 1.1% compared with the manual measurement error. Mu et al. (2020) combined R-CNN and ResNet101 to design a green tomato detection model and compile the location map of tomatoes to achieve the detection, counting, localization, and size estimation for tomato ripeness detection and yield prediction. Gan et al. (2020) designed a thermal imaging rig to capture images of immature citrus and build a deep learning model for fruit counting by the temperature difference feature between the object fruit and the background. Jia et al. (2020b) improved the instance segmentation model Mask R-CNN to adapt the detection of apple targets by combining ResNet and DenseNet as the feature extraction network of the original model, which significantly improved the detection accuracy of apple targets in overlapping and branch-obscured environments. However, Mask R-CNN segmentation takes a long time and cannot meet the real-time requirements of segmentation. The above recognition models have substantially improved in terms of accuracy and robustness compared with traditional vision methods. However, in the complex orchard environment, the real-time operation capability of agricultural equipment needs to be further improved. Fruit detection and segmentation are essential for future agronomic management. The research method YOLOF-snake focuses on the rapid identification and segmentation of green fruit. It uses a detection method that only one layer of feature maps is used and a segmentation module that iteratively adjusts the contour of the target fruit. Fusion can reduce the running time of the current mainstream fruit segmentation method by about half. Different from other models, only the fifth-level feature map extracted from the backbone network is used. The fifth-level feature map not only contains enough context information to detect targets of various scales but also shortens the operation of the model detection time. In addition, the model is compared with other mainstream models, and the comparison revealed that the running time of the model is greatly reduced, while accuracy is guaranteed. It is being applied to the vision system of fruit and vegetable picking robots and can also greatly improve the efficiency of fruit and vegetable picking robots. At the same time, since most crop fruits are green during growth, this method can also be applied to the identification and segmentation of other green fruit, such as immature persimmons, immature tomatoes, cucumbers, green peppers, and other crops. Therefore, the method can accurately count the growth cycles of these fruits and simultaneously perform proper variable rate irrigation and fertilization on the monitored growth state or density of the fruits at each stage. At the same time, it improves the efficiency of resource utilization and the quality of the final ripe fruit. In addition, this method can also provide an essential reference for the production estimation of farm operations, fruit trade, retailers, and storage facilities. By detecting and quantifying the distribution of canopy fruits, farmers can obtain valuable information and provide references for optimizing these processes, significantly promoting the temporal and spatial management of agricultural production. As the most basic and vital part of agricultural robots, the vision system is used to analyze the specified targets from complex and diverse scenes, directly affecting the quality and efficiency of fruit picking. The design of the vision system aimed at rapid positioning and precise segmentation will significantly affect the real-time and reliability of the actual application of harvesting robots.

## Data Sample Collection and Pre-Processing

**FIGURE 1:**
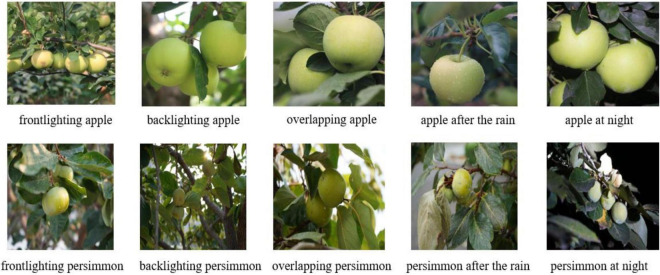
Images of apple and persimmon in front light, backlight, overlap, after rain, and night.

The images of green apples and persimmons are collected at the Shouguang Agricultural Demonstration Base using a Sony Alpha 7 camera with a resolution of 6000 × 4000. To facilitate the contrast experiments, the original size of 4000 × 4000 pixels is cropped and further reduced to 512 × 512 pixels. Close-up and long-range images are collected to show the adaptability of the model to large and small object fruit, and images of apples and persimmons are also collected under different shading conditions. To obtain accurate results, the images are also acquired in both front-lighting and back-lighting conditions. LabelMe data labeling software is used to label these images. Most miniature outer rectangular boxes of green apples and persimmons are used as ground-truth bounding boxes to reduce the interference of background factors during detection. Figure 1 shows the green apple and persimmon fruits images that are collected under different lighting conditions.

As can be seen from Figure 1, blurred fruit boundaries in overlapping fruits, weak backlight light intensity on the fruit surface, and water droplets on the fruit surface after rain all become factors that affect the accuracy of green fruit recognition and segmentation. For the collection of green fruit images, various factors that may affect fruit recognition and segmentation in the complex orchard background are fully considered. The image samples are collected with maximum consideration of the complex actual environment of the orchard, and 568 persimmon images and 515 apple images are collected and classified into a variety of situations, including overlapping, direct light, after rain, backlight, and night. The details of the sample distribution are shown in Table 1.

**TABLE 1:** Sample distribution details of green fruit images.

Condition	Overlapping	Direct sunlight	After the rain	Backlight
Number of persimmon images	523	109	113	207
Number of apple images	459	89	103	265

## YOLOF Object Detection Model

### Use of Single-Layer Features

**FIGURE 2:**
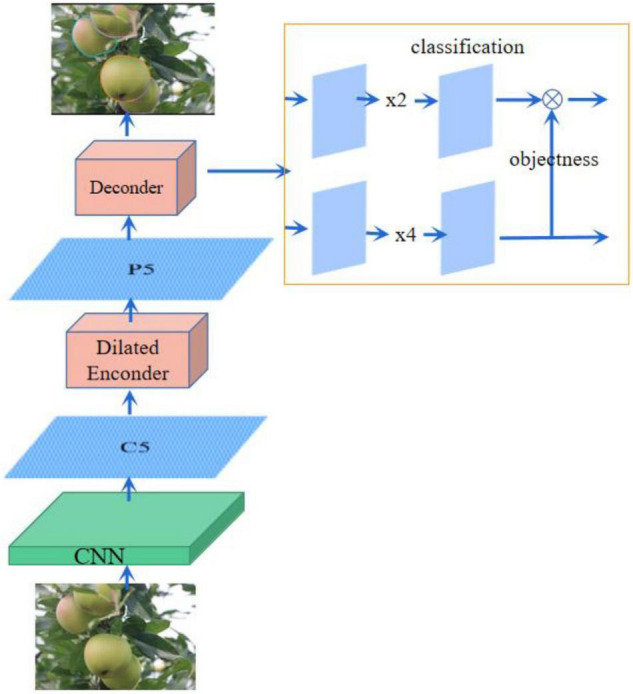
YOLOF structure schematic diagram.

The YOLOF-snake is presented in this article, a fruit segmentation model based on the YOLOF object detection method. The YOLOF algorithm is a rethinking of FPN for single-stage object detection and shows that the success of FPN lies in its separate processing ideas rather than feature fusion ideas. Its outstanding features are fast and accurate. Unlike the object detection models using FPN networks, YOLOF is a simple object detection framework that uses a layer of feature maps. The original ResNet101 network is used, and after the green fruit features are extracted, only one layer of the feature map is used, with an inflated encoder and an equilibrium matching strategy added for object fruit detection. The object fruit class and ground truth are predicted. Three components are included: the backbone network, the encoder, and the decoder. The structure is shown in Figure 2.

### Large-Scale Variation

For the detection of green fruit, it is a difficult task to detect the object fruit with a large-scale variation. Since only one layer of feature map is used in this method, a restricted sensory field can only be covered by one layer of features, and the mismatch between the sensory field and the object scale will make the detection effect poor. To address this problem, YOLOF first increases the receptive field by stacking the standard convolution and expanding the convolution, but it still cannot cover all the object scales. However, if the original feature map and the feature map after extending the receptive field are integrated using the residual linkage and construction extension module, then in this case, the feature maps of all target scales can be obtained. Finally, feature maps covering all scales of the target fruit can be achieved.

## YOLOF-Snake for Green Fruit Segmentation

The YOLOF-snake algorithm completes target fruit segmentation based on the original YOLOF target fruit detection method, enabling the fruit picking robot to pick green fruits accurately and quickly. YOLOF is an object detection model in which only a 32-fold downsampled C5 feature map is used. The residual module is used to extract multi-scale contextual features for objects at different scales, and the deficiency of multi-scale features is compensated. A balanced matching mechanism is used to solve the positive sample imbalance problem caused by sparse anchors in the single feature map. YOLOF can demonstrate the good results of green fruit recognition, and the real-time performance and accuracy of the green fruit picking work of the picking robot are improved. Therefore, YOLOF is used to implement green fruit recognition, green fruit regression boxes are obtained and fed into an additional snake network, and green fruit segmentation is implemented.

### The Overall Structure of YOLOF-Snake

**FIGURE 3:**
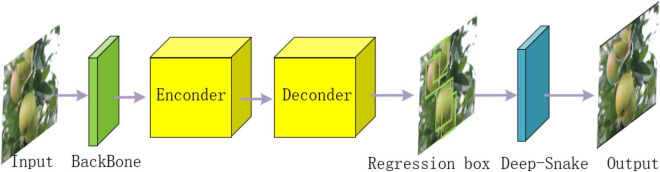
Overall structure of YOLOF-snake. 1. Output the feature map based on the underlying backbone. 2. Enter the encoder. 3. Decoder to obtain the classification results and regression box. 4. Deep-snake network to segment the regression boxes for the object fruit.

Figure 3 illustrates the overall structure of YOLOF-snake, which consists of four parts.

#### Backbone Network

The acquired dataset images are fed into the backbone ResNet101 to extract features, and several layers of feature maps are output. Since the fifth layer feature map contains sufficient contextual information for detecting targets of various scales, the fifth layer feature map with a channel count of 2048 and a downsampling rate of 32 is selected.

#### Encoder Structure

**FIGURE 4:**
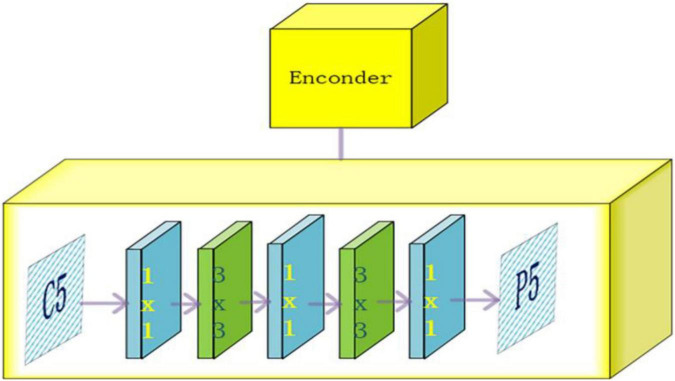
Encoder structure diagram.

The output C5 layer feature map is used by the encoder to expand the receptive field, matching the sensory field to the target scale. Two modules are included, the projection layer module and the residual module. The projection layer module consists of two parts, a 1 × 1 convolutional layer is used to reduce the size of the channels and a 3 × 3 convolutional layer, is used to refine the semantic information of the context to obtain a feature map of 512 channels. The residual module generates output features with multiple receptive fields that can cover all scales of the target fruit. The residual module superimposes four consecutive residual blocks with different convolution kernel expansion rates, each consisting of three convolutions. The first layer is a 1 × 1 convolution, after the first convolution channel is reduced to 128, then a 3 × 3 convolution expansion convolution is used to increase the receptive field, and finally, the channel size is recovered by a 1 × 1 convolution. The specific structure is shown in Figure 4.

#### Decoder Structure

The decoder classifies and regresses the output of the encoder and then inputs the regression box into the Deep-snake network for target fruit segmentation. The decoder contains two branches, similar to the classification and regression branches of RetinaNet. To achieve better recognition, it has two improvements. First, the different numbers of convolutional layers in the regression branch and classification branch are set, in the regression branch, four convolutional layers plus batch normalization and activation function layers are used, while in the classification branch two convolutional layers are set for regression. Second, an implicit object indicator is added to each anchor of the regression branch, and the final classification result is the result of multiplying the output of the classification branch and the score of the object indicator.

#### Segmentation Model

The regression box is taken to the midpoints, and a diamond shape is obtained after connecting the midpoints. Deep-snake network performs offset prediction for four points, the object is the extreme value point around the object, and the center of the extreme value point is extended uniformly to the two directions of the edge where the extreme value point is located. The contour is adjusted iteratively by Deep-snake until it overlaps with the boundary of the object fruit.

### Deep-Snake Instance Segmentation Method

As the YOLOF method only detects and classifies the object fruit, it cannot accurately determine the location of the object fruit, which makes the green fruit picking robot cannot accurately and quickly carry out the picking process. Therefore, the contour-based instance segmentation method Deep-snake algorithm module is embedded after the YOLOF regression branch to achieve the segmentation results of the object fruit. It allows the green fruit picking robot to locate the object fruit quickly and accurately and has more effective results of occluded and overlapping fruits.

**FIGURE 5:**
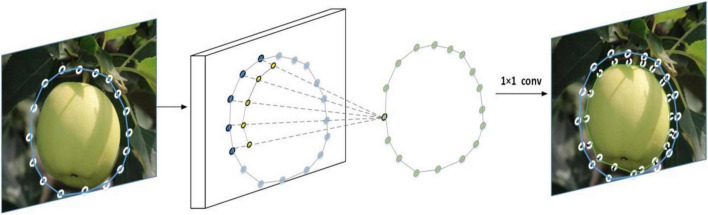
Fine-tuning of the profile of the apple fruit.

First, the regression box of the target fruit is obtained by the YOLOF method before inputting the Deep-snake module, and the regression box is gradually optimized as the boundary of the target fruit to carry out the segmentation of the target fruit. Second, for the learning of vertex features, the circular convolution is used to implement the topology, which helps to optimize the contours of green fruits, and to prevent the Deep-snake algorithm from falling into a local optimum solution, how to fine-tune the contours being learned directly from the data, as shown detail in Figure 5.

In traditional instance segmentation methods, the vertex coordinates are treated as variables to optimize the manually designed energy function and the target boundary is fitted by minimizing the energy function. The energy function is usually concave and requires manual design based on low-dimensional image features, which usually leads to locally optimal solutions. Instead, learning how to fine-tune the contours directly from the data is chosen, for N vertices {Xi i = 1,…., N}, the feature vector for each vertex is first constructed, and the feature F of vertex Xi is the function [F(Xi); Xi’] corresponding to the network features and vertex coordinates, where F is the feature map of the backbone output, F(xi) is the bilinear difference output at vertex Xi, the additional Xi is used to describe the location relationship between the vertices, Xi is translation invariant, and the relative coordinates are obtained by the coordinates of each vertex subtracting the minimum x and y of all vertices in the contour.

After the feature vectors of the vertices are obtained, further learning of the features of the contour is required. The features of the vertices can be treated as a one-dimensional discrete signal f: Z→R^D^, and then, the vertices are processed one by one. In order not to destroy the topology of the contour, a periodic signal is extended and is defined as follows:


(1) $${\left(f_{N}\right)}_{i}≜\sum_{j=-\infty }^{\infty }f_{i-j\,N}=f_{i\,\left(\text{mod}\,N\right)}$$

The circular convolution of Eq. 2 is applied for feature learning.


(2) $${\left({\text{f}}_{\text{N}}*k\right)}_{\text{i}}=\;{\sum_{\text{j=-r}}^{\text{r}}\left({\text{f}}_{\text{N}}\right)}_{\text{i+j}}{\text{k}}_{\text{j}}$$

The periodic signal of Eq. 1 is the definition of the vertex feature, k: [−r,r]→R^D^ is the learnable convolution kernel, and * is the standard convolution operation in Eq. 2. The graph representation is similar to the standard convolution operation and can be integrated into the current network quite simply. After feature learning, three 1 × 1 convolution layers are used by each vertex to offset the output, and the size of the cyclic convolutional kernel is 9 in the experiment.

### Details of the Structure of the Example Segmentation Model YOLOF-Snake Network

**FIGURE 6:**
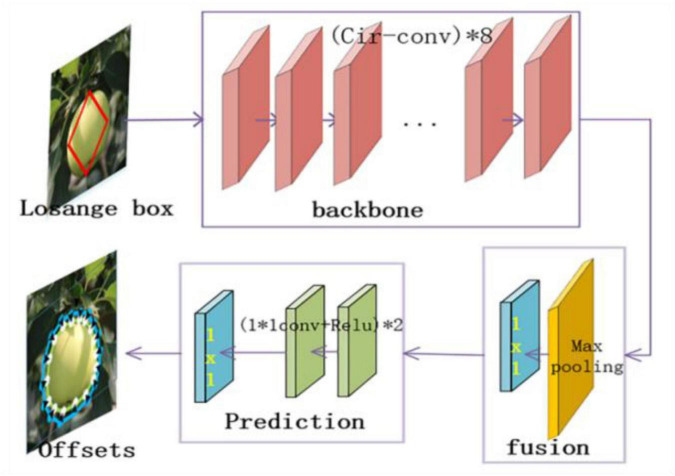
Network structure of the add-on module.

The Deep-snake is added to the YOLOF object detection model for instance segmentation. First, the target box is generated by YOLOF, the midpoints of the target regression box are connected to form a rhombus box, and then, the Deep-snake algorithm is used to adjust the vertex target poles of the rhombus to form an octagonal contour. Finally, the Deep-snake algorithm is used to iteratively adjust to obtain the target shape. For the extraction of the initial contour, the idea of poles is adopted, after the rectangular regression box is obtained, and the centroids of the four edges are obtained $\left\{x_{i}^{e\,x}\,i=1,2,3,4\right\}$. For each polar point, the 1/4 of the length of the regression box in two directions along the box is expanded, and if it exceeds the range of the original box, it is stopped. Finally, several extended edges are added, and the octagon is formed. After that the octagon and the object edges of N points are sampled equally, where the sampling of the object edges is the ground-truth contour sampling the object edges equally for N points, N is 128, and the point of the upper pole $x_{1}^{e\,x}$ will be the starting point. The 40 points are sampled equally before the diamond contour is input into the Deep-snake. If the vertices are far away from the ground-truth bounding box, the adjustment becomes more difficult. Therefore, an iterative method is used to perform Deep-snake adjustment with three iterations. The backbone contains eight circular convolutional layers, each using residual connections. Fusion block is used to fuse the multi-scale contour features in the backbone network, containing one 1 × 1 convolutional layer and one maximum pooling layer. The prediction branch uses three 1 × 1 convolutions to output each vertex’s offset. The specific network structure is shown in Figure 6.

### Selection of Positive and Negative Samples

The YOLOF method for green fruit recognition is an anchor-based method. For the selection of anchor box, each pixel point has three anchor boxes with different aspect ratios {1:2, 1:1, and 2:1}. To cover the fruit object more effectively, three sizes of anchor boxes are set for each aspect ratio as follows {2^0^, 2^1/3^, and 2^2/3^}; thus for each pixel point, there will be nine anchor boxes. Each anchor box is associated with a K-dimensional vector, where K is the number of categories of the target fruit and a four-dimensional vector for the border regression. In general, the definition of the positive sample is based on the Intersection over Union (IoU) between the anchor box and the ground truth. In RetinaNet, anchor boxes are associated with ground truth when the IoU is greater than 0.5; IoU in (0, 0.4) is used as background; each anchor box is associated with a maximum of one ground truth. In the K-dimensional vector, the associated category value is 1, while others are 0. Anchor boxes with IoU between (0.4 and 0.5) are discarded. The border regression is to calculate the offset between the anchor box to the associated ground-truth bounding boxes. However, for our proposed structure, the number of anchor boxes is much reduced and very sparse because only one layer of features is used. Using the above matching strategy for sparse anchor boxes causes the problem that large ground truth will generate more positive anchor boxes than small ground truth, thus causing the imbalance problem of positive anchor boxes, which will cause the YOLOF target detector to focus only on large targets and ignore small targets.

A balanced matching strategy is used to solve the above problem. For each ground-truth bounding box, only the closest k anchor boxes are used as positive anchor boxes, so that the ground-truth bounding boxes of different sizes will have the same number of positive anchor boxes, ensuring that all ground truth can participate in training with the same probability. Meanwhile, the negative samples with IoU greater than 0.7 and the positive samples with IoU less than 0.15 are ignored, so that the negative samples with large IoU and the positive samples with small IoU are filtered out.

### Loss Function

The loss function is used to estimate the degree of inconsistency between the predicted and true values of our model, which determines the effectiveness of our model for the fruit detection and segmentation. The loss function contains four components, the loss function is *L*_*YOLOF*_ in the detection module, and the loss function *L*_*snake*_ in the segmentation module consists of the loss function *L*_*ex*_ for the poles and the iterative contour adjustment loss function *L*_*iter*_. In addition, the loss function of the detection module includes the loss function of the classification and the loss function of the regression boxes. The focal loss function is applied for the loss function of the classification*L*_*cls*_. The experiments show that γ = 2 works best and the robust interval is γ∈ [0.5, 5]. Besides, the Smooth-L1 loss function is employed for the loss function of the border regression L*res*. So the overall loss function is as follows:


(3) $$L_{y,s}=L_{Y\,O\,L\,O\,F}+L_{s\,n\,a\,k\,e}=L_{c\,l\,s}+L_{r\,e\,s}+L_{e\,x}+L_{i\,t\,e\,r}$$

Where,


(4) $$L_{c\,l\,s}=f\,\left(x\right)=\left\{ \begin{array}{c} -\alpha \,{\left(1-y\right)}^{\gamma }\,l\,o\,g\,y^{\prime }\\ -\left(1-\alpha \right)\,y^{\prime }\,l\,o\,g\,y \end{array}\right.$$

(5) $$L_{r\,e}=f\,\left(x\right)=\{\begin{array}{cc} 0.5\,{\left(\sigma \,x\right)}^{2}, & i\,f\,\left|x\right|<\frac{1}{\sigma^{2}}\\ \left|x\right|-\frac{0.5}{\sigma^{2}}, & o\,t\,h\,e\,r\,w\,i\,s\,e \end{array}$$

(6) Le⁢x=14∑i=14ℓ1(xe⁢x∼i-xie⁢x)

(7) Li⁢t⁢e⁢r=1N∑i=1Nℓ1(x-∼ixig⁢t)

where xex∼i is the predicted extreme value points, and xi∼ is the deformed contour point, which is the boundary point of the true contour of the fruits. α = 0.25, γ = 2, and σ = 3 are default values.

## Experimental Results

**FIGURE 7:**
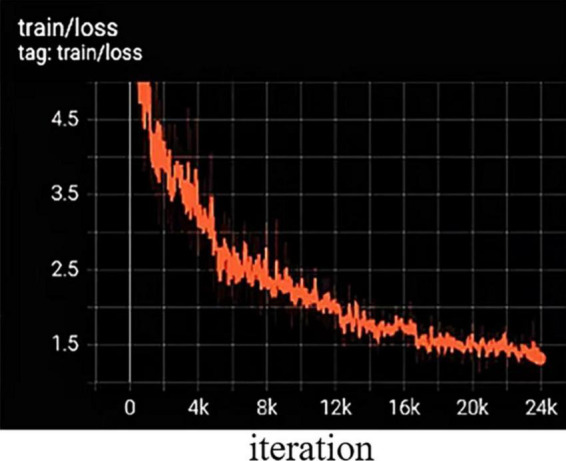
Training error variation curve.

To obtain fast and accurate segmentation results for green fruit in complex environments, the loss function is optimized to balance the ground truth, confidence, and error of segmentation results. The trend of the loss function can be represented by the training error curve in Figure 7, and the training and validation sets are trained for about 24 iterations. The network is fitted quickly in the first six iterations, and the loss function stabilized after 16 iterations.

In addition, the model is pre-trained and the optimal training model is selected to test the segmentation of green fruits on the validation set to ensure the real-time performance and accuracy of the model. Finally, the detection and segmentation effects of the method and more advanced instance segmentation methods for green fruits are compared. The average precision (AP) and detection time (T) are used as the main performance evaluation metrics.

### Experimental Platform and Details

The experiments are run on a server with Ubuntu 18.04 operating system, 64 GB of running memory, an NVIDIA 2060Ti graphics card with 32 GB of video memory, and a CUDA 10.0 environment. The experiments are based on the PyTorch deep learning framework, a virtual environment of python 3.7 on Anaconda, with an input image of 640 × 480 pixels and an initial learning rate of 0.0025.

The following section describes the details of the implementation.

The detection part: First, ResNet101 is employed as the backbone network to extract the features of the image, and after the extraction of the green fruit features, a fifth layer feature map with 32 times downsampling and 2048 number of channels is selected. After that, four identical residual blocks are employed to process the feature maps, and there are two branches to perform classification and boundary regression for output. Then, the negative samples with IoU less than 0.7 and positive samples with IoU greater than 0.15 are selected and a loss function based on stochastic gradient descent is used for training.

The segmentation part: First, the regression box of the target fruit obtained from the YOLOF model is optimized to the contour of the target fruit. The segmentation module used a recurrent convolutional network to iteratively adjust the contour until it is coincident with the boundary of the target fruit. The network of the segmentation module is iterated three times, and the results of each iteration are saved. Finally, the results of the three iterations are used to evaluate the YOLOF-snake segmentation model.

### Evaluation Metrics

To evaluate the effectiveness of our model for green fruit segmentation, the average precision AP and time T are applied, where the calculation of AP entailed first calculating true cases (TP), false-positive cases (FP) based on the IoU, followed by ranking the confidence of each ground truth from high to low, obtaining the precision P and recall R. The value of AP is calculated after plotting the PR curve. The average of the ten is taken as mean average precision (mAP) (mAP = 1/10∑_*i* ∈ *I*_AP_*IOU* = *i*_). mAP can comprehensively measure P, R, and threshold; thus, it can be strongly persuasive when evaluating the model. The equations for P, R, and AP are as follows:


(8) $$P=\frac{T\,P}{T\,P+F\,P}$$

(9) $$R=\frac{T\,P}{T\,P+F\,N}$$

(10) $${\text{AP}}_{IoU=i}=\;1/101\sum_{r\in R}p(r)=\;1/101\;\sum_{r\in R}{\text{max}}_{\tilde{r}:\tilde{r}\geq r}p(\tilde{r})$$

Where, TP—the number of samples that are actually positive and are detected as such.

FP—the number of samples that are actually negative and detected as positive.

FN—the number of samples that are actually negative and are detected as such.

i—threshold in IoU: [0.5, 0.55, 0.6,…, 0.95].

r—recall, the precision associated with recall.

R—[0, 0.01, 0.02,… 1.0] with an interval of 0.01 and a total of 101 values.

The ground truth obtained after the non-maximum suppression (NMS) method may not be the correct category, so a ground truth with a confidence level greater than a threshold of 0.5 is defined as a positive sample and vice versa; a positive sample with an IoU greater than a threshold of 0.6 with the ground-truth bounding box is considered a TP and vice versa as an FP. FN is the presence of an actual positive sample in a negative sample.

### Results and Analysis

#### Model Training

**FIGURE 8:**
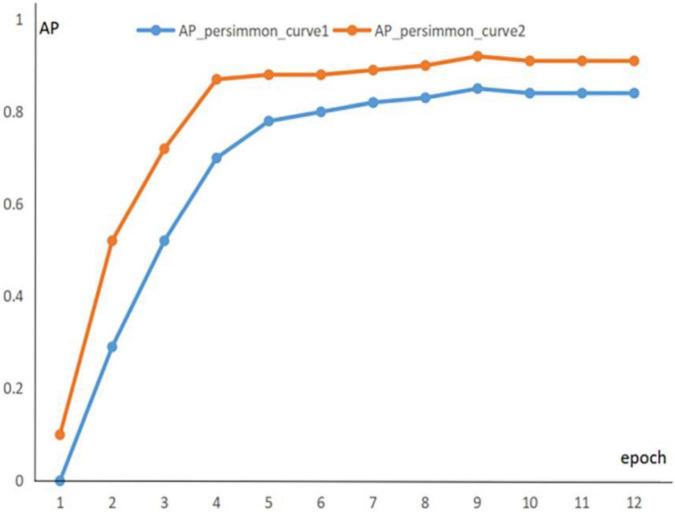
AP evolution curve of persimmon dataset.

**FIGURE 9:**
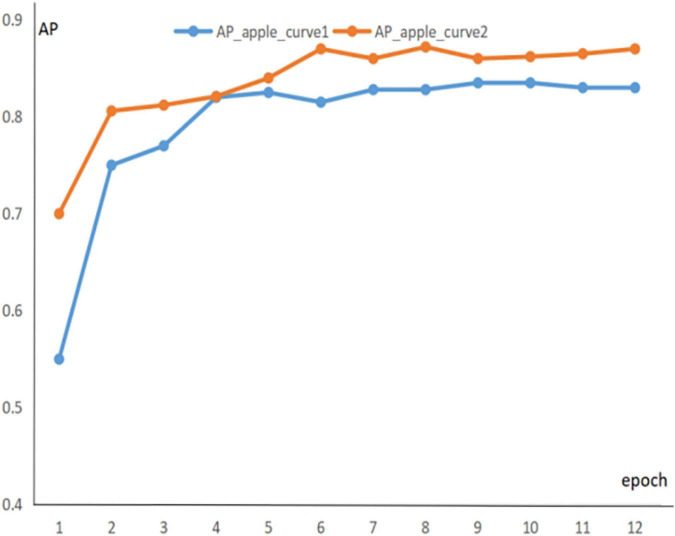
AP evolution curve of apple dataset.

To prevent experimental overfitting from occurring, pre-training is used to provide a larger number of training samples when training the green fruit dataset, allowing for a faster fit. The 1600 images containing apples are extracted from the common COCO dataset, these images are applied for pre-training experiments, and the information of the pre-trained parameters is recorded. Then, the dataset is trained and the pre-trained parameters are transferred to the formal training as initial weights. It is found that pre-training significantly improved the segmentation of the model. Figures 8, 9 show the segmentation AP for the apple and persimmon datasets with and without the pre-training method, respectively.

The blue curve in the figure indicates the AP curve for segmentation of green fruit with random initial weights, and the orange curve indicates the AP curve for segmentation of green fruit with pre-training parameters as initial weights. The above line graph shows that the orange curve is significantly higher than the blue curve for the segmentation AP of green fruit, which indicates that the segmentation accuracy and generalization ability of the model is improved by pre-training experiments, benefiting the model to be better applied to the segmentation of other green fruits.

#### Segmentation Effect

**FIGURE 10:**
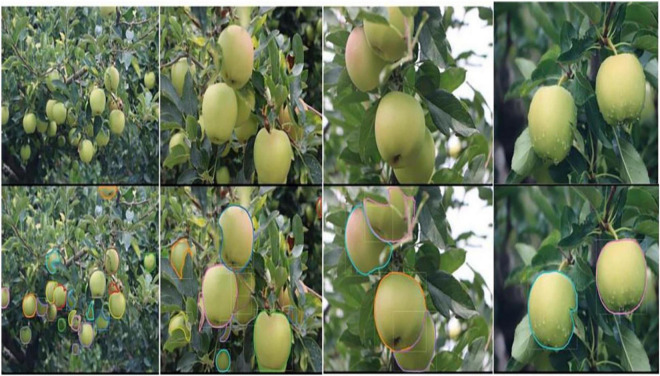
Green apple result images.

**FIGURE 11:**
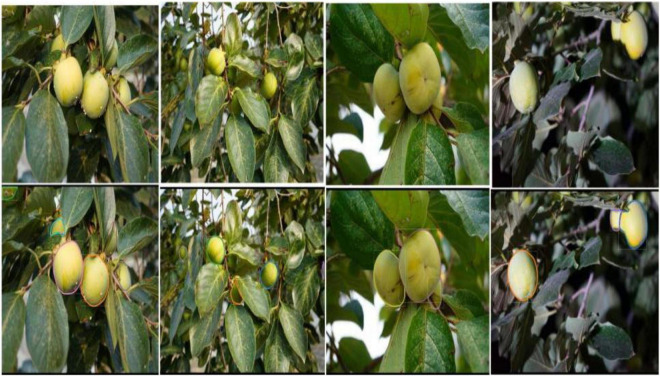
Green persimmon result images.

In the actual complex orchard background, the segmentation results of the fruits are affected by overlapping, blocking, and lighting factors, so the segmentation difficulty for each case is different. Overlapping obscured green fruits are more difficult to segment, while images with a smaller number of fruits and a simple background are easier to segment and produce better results. Segmentation of green fruit is also more difficult in backlight and at night. The results of YOLOF-snake model for green fruit segmentation are shown in Figures 10, 11.

In each pair of images of the segmentation effects, the top and bottom are the original images input to the model and the segmented result images, respectively. As can be seen, the green fruit against the same colored background can still be segmented without segmentation errors or missed detection errors. The target fruits whose edges are obscured by branches or overlapping fruits can also be accurately segmented. This indicates that the model has a good generalization capability and good resistance to interference.

**FIGURE 12:**
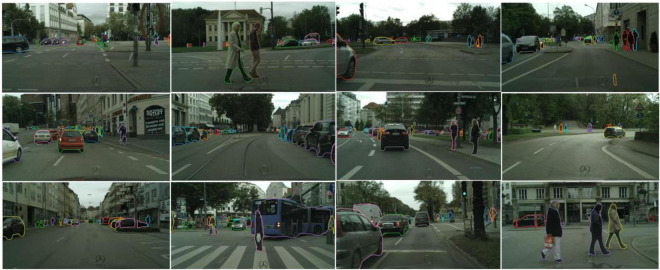
Result images of the Cityscapes data set.

At the same time, to verify the validity of the experimental results, the public dataset Cityscapes is chosen to conduct experiments. Experiments show that YOLOF-Snake can achieve high accuracy and good results on the public dataset. The following Figure 12 shows some result images.

### Ablation Experiments

Ablation experiments are carried out to analyze the residual block count problem in the YOLOF structure, and comparisons of the experiments are shown in Table 2.

**TABLE 2:** Number of residual blocks.

Number	mAP/%	mAP_*s*_/%	mAP_*m*_/%	mAP_*l*_/%
0	62.3	47.6	79.5	72.5
2	63.5	47.6	70.1	75.2
√4	64.9	47.8	70.2	77.3
6	65.0	47.6	70.2	78.2
8	66.1	48.5	71.0	79.1
				

As described in Table 2, the segmentation efficiency for green fruit is improved by changing the number of residual blocks, with the accuracy increasing with the number of residual blocks. However, the four residuals in the encoder are added to keep the model simple. Through ablation experiments, the residual blocks are applied to the model to obtain better segmentation results for green fruit and to deal with problems, such as fruit overlap and branch occlusion.

### Comparisons

As the model is a two-stage approach to green fruit recognition and segmentation, the accuracy of recognition and segmentation is compared with other better-performing object detection and segmentation methods, respectively. Since the main feature of the model is simplicity and efficiency, it is also compared with other methods in terms of time. The current advanced and effective object detection and segmentation methods are collected. The same experimental platform configuration is utilized, and the evaluation results are shown in Table 3.

**TABLE 3:** Performance comparison of detection and segmentation methods.

Methods	*Appledataset*		*Persimmondataset*		Average time/s
		
	mAP	*mAP^S^*	mAP	mAP *^S^*	
SOLO	—	57.4	—	76.5	0.49
PolarMask	56.3	53.8	68.3	66.1	0.52
YOLACT	57.6	60.5	66.9	71.2	0.45
TensorMask	—	65.3	—	72.4	0.71
FCOS	61.2	—	78.6	—	0.42
RetinaMask	69.2	69.4	77.5	73.1	0.92
*Mask*R-CNN	70.3	70.9	79.9	79.3	0.48
*MS*R-CNN	71.2	71.6	80.3	80.9	0.56
OURS	71.0	71.4	81.8	82.6	0.23
					

Table 3 shows comparisons of the recognition and segmentation accuracy of several advanced segmentation methods for apples and persimmons, respectively. The table shows the records of recognition and segmentation accuracy for green apples and green persimmons, respectively. “——” indicates that the method has no detection or segmentation ability.

As can be seen from Table 3, the method has the highest accuracy with better detection of apples and persimmons. Although the segmentation accuracy for apples is 0.2% lower than that of MS R-CNN (Huang et al., 2019), YOLOF-snake has a cleaner structure, faster detection speed, and smaller computation than MS R-CNN, enabling real-time and accurate detection of green fruits in a complex orchard background. A comparison of the detection times of the various models and the method is shown in Table 3. Through the above comparison and analysis, the method performs more outstandingly in terms of accuracy and real-time, which improves the efficiency of the work of the orchard picking robot and gives some practical significance to the solution of the problem of branch and leaf shading and fruits overlapping.

## Conclusion

The YOLOF algorithm is applied by YOLOF-snake for the segmentation of green fruit in complex orchard backgrounds. The Deep-snake method is added to the YOLOF object detection model for the segmentation of green object fruit for branch shading and overlapping fruit, and poor light intensity at night and back-lighting in the complex orchards. The four residual blocks of YOLOF are used to solve the problem of restricted perceptual field. After the regression box of the object fruit is obtained, the Deep-snake network performs offset prediction on the four midpoints of the regression box, taking the extreme points around the object as the object and the center, extending uniformly in both directions to the boundary where the extreme points are located. The segmentation module then iteratively adjusts the contour lines until they coincide with the boundaries of the object fruit. Simultaneous circular convolution is used to implement vertex feature learning for the green object fruit to achieve a more desirable segmentation result for the green object fruit. The segmentation accuracy for apple fruits in the complex orchard backgrounds is 71.4%, and the segmentation accuracy for persimmon is 82.6%. Compared with MS R-CNN, although YOLOF-snake achieves similar results to MS R-CNN in terms of segmentation accuracy, the computational volume and complexity of the model are smaller and the network structure is simpler. While comparing with other network models, the model improves the recognition accuracy and speed. Overall, the model has the best detection performance.

The efficiency of orchard harvesting robots has been further improved by targeting the occlusion and overlap of green fruits in complex orchards. In pursuit of better fruit picking and fruit and vegetable production, other factors affecting fruit detection and segmentation should be considered in future research. Moreover, the network structure should be optimized by combining the ideas of lightweight networks and feature fusion to obtain better recognition and segmentation of green orchards.

## Data Availability Statement

The original contributions presented in the study are included in the article/supplementary material, further inquiries can be directed to the corresponding authors.

## Author Contributions

WJ: conceptualization, data curation, and writing – original draft preparation. ML: methodology, visualization, and writing – original draft preparation. RL: investigation and writing – reviewing and editing. CW: software and validation. NP: visualization and validation. XY: investigation, software, and validation. XG: conceptualization and writing – reviewing and editing. All authors contributed to the article and approved the submitted version.

## Conflict of Interest

The authors declare that the research was conducted in the absence of any commercial or financial relationships that could be construed as a potential conflict of interest.

## Publisher’s Note

All claims expressed in this article are solely those of the authors and do not necessarily represent those of their affiliated organizations, or those of the publisher, the editors and the reviewers. Any product that may be evaluated in this article, or claim that may be made by its manufacturer, is not guaranteed or endorsed by the publisher.
